# Experimental increase of worker diversity benefits brood production in ants

**DOI:** 10.1186/s12862-021-01890-x

**Published:** 2021-08-30

**Authors:** Marina N. Psalti, Dustin Gohlke, Romain Libbrecht

**Affiliations:** Institute of Organismic and Molecular Evolution, Johannes Gutenberg University of Mainz, Hanns-Dieter-Hüsch-Weg 15, 55128 Mainz, Germany

**Keywords:** Social evolution, Social insects, Division of labor, Behavior, Genetic diversity

## Abstract

**Background:**

The reproductive division of labor of eusocial insects, whereby one or several queens monopolize reproduction, evolved in a context of high genetic relatedness. However, many extant eusocial species have developed strategies that decrease genetic relatedness in their colonies, suggesting some benefits of the increased diversity. Multiple studies support this hypothesis by showing positive correlations between genetic diversity and colony fitness, as well as finding effects of experimental manipulations of diversity on colony performance. However, alternative explanations could account for most of these reports, and the benefits of diversity on performance in eusocial insects still await validation. In this study, we experimentally increased worker diversity in small colonies of the ant *Lasius niger* while controlling for typical confounding factors.

**Results:**

We found that experimental colonies composed of workers coming from three different source colonies produced more larvae and showed more variation in size compared to groups of workers coming from a single colony.

**Conclusions:**

We propose that the benefits of increased diversity stemmed from an improved division of labor. Our study confirms that worker diversity enhances colony performance, thus providing a possible explanation for the evolution of multiply mated queens and multiple-queen colonies in many species of eusocial insects.

**Supplementary Information:**

The online version contains supplementary material available at 10.1186/s12862-021-01890-x.

## Introduction

Genetic relatedness plays an important role in the evolution of altruistic behaviors in animals [1]. Extreme altruism is found in colonies of eusocial Hymenoptera (ants, bees and wasps), where the workers forgo their own reproduction to help the queens produce offspring [2]. Such reproductive division of labor evolved in a context of high genetic relatedness, with a single female reproductive mated with a single male [3, 4]. Most extant eusocial Hymenoptera species are still characterized by high genetic relatedness [3].

Other species evolved colonies with lower relatedness among individuals, and thus higher genetic diversity [5]. In these species, colonies have one multiply mated queen and/or multiple queens. Prominent examples include the honeybee *Apis mellifera*, where queens can mate with up to 20 males [6–9], and the Argentine ant *Linepithema humile*, where nests may contain up to 60 queens [10]*.* However, there are costs associated with strategies that increase genetic diversity. Multiple mating increases risk of disease or predation, and requires more energy to locate the sexual partners and copulate [11, 12]. Having multiple queens per nest lowers relatedness in the worker force and may favor the emergence of conflicts among workers [13–17].

The evolution of such strategies to increase genetic diversity in some eusocial insect species shows that they must have benefits in certain ecological conditions [18]. The potential benefits of increased genetic diversity include increased resistance to diseases and parasites via improved social immunity [19–27] and a more efficient behavioral division of labor among workers [28–33].

Behavioral division of labor is the repartition of tasks in the worker force. For example, some workers tend to stay inside the nest to nurse the brood while others forage for food. These tendencies likely stem from workers differing in their internal response threshold to perform specific tasks [34]. This response threshold is determined by a combination of extrinsic and intrinsic factors, such as the social environment, location in the nest, morphology, age, individual experience and genetic background [35–42]. The increasing evidence for genetic effects on worker behavior and division of labor [29–33, 43–45] is consistent with the hypothesis that increased genetic diversity in the worker force would result in a larger variation in threshold responses, and thus a more efficient division of labor.

Several lines of evidence suggest that intracolonial genetic diversity increases fitness. The reports of such findings fall in one of three categories. First, there are theoretical studies that supported a link between diversity and performance [17, 46–48]. Second, there are reports of correlations between genetic diversity and one or several fitness correlates in several species of bees, wasps and ants [28, 49–57]. Third, there are reports of experimental manipulations of genetic diversity that affected colony performance, mostly in bees [19, 20, 23, 24, 38, 51, 58–61].

However, there is still debate over whether increased genetic diversity directly benefits colony performance [62]. First, finding correlations between genetic diversity and fitness components does not imply causation, and other correlative studies did not detect such an association [49, 53, 63–65]. Then, the strategy of many studies that experimentally manipulated genetic diversity was to decrease it in species with naturally high diversity. For example, in the highly polyandrous honey bee, the artificial insemination of queens with the sperm from a single male reduced the performance of their colonies compared to queens inseminated with the sperm from multiple males [38, 51, 61]. In these studies, the decrease in colony performance associated with the low diversity treatment could be confounded by potential stress associated with not being in the natural state. Two studies in *Bombus terrestris* showed some benefits of artificially increased genetic diversity in a species with naturally lower diversity, but mostly in terms of resistance to pathogens [59, 60]. Finally, experiments based on artificial insemination cannot disentangle between direct effects of genetic diversity among workers produced by the artificially inseminated queen and indirect maternal effects via the queen (e.g., on the number and quality of eggs produced) in response to the insemination with variable sperm diversity.

One way to get around the confounding maternal effects is to directly manipulate the diversity in the worker force. This experimental approach has so far been restricted to the study of the effect of worker diversity on pathogen resistance in bumble bees [20] and ants [23]. Here, we experimentally increased worker diversity in small colonies of the black garden ant *Lasius niger*, while controlling for potential maternal effects. We produced colonies composed of workers from either one (low diversity) or three (high diversity) source colonies. These experimental colonies were then provided with a single, unrelated queen, and brood production was monitored over time. We found an increased brood production in experimental colonies with a more diverse worker force, thus showing that worker diversity enhances colony performance.

## Results

### The experimental increase in worker diversity enhanced the production of larvae, but not eggs

**Fig. 1:**
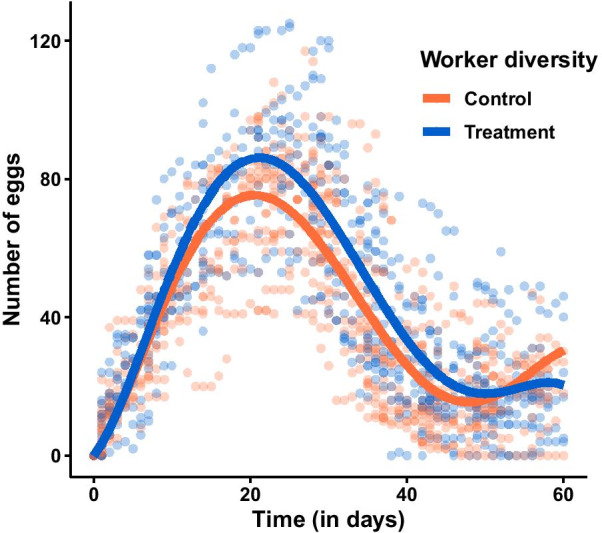
Number of eggs in control (n = 23) and treatment (n = 18) colonies over time. The dots show the raw data for all colonies and time points. The curves depict the output of the models for control (orange) and treatment (blue) colonies. None of the parameters of the quintic function differed significantly between control and treatment colonies (all p ≥ 0.1; Table 1)

To measure a potential effect of worker diversity on offspring production we monitored the number of eggs and larvae in experimental colonies with low (control) and high (treatment) worker diversity. The change over time in the number of eggs recorded in the experimental colonies did not differ between control (n = 23) and treatment (n = 18) colonies (p ≥ 0.1 for all parameters; Table 1; Fig. 1). Consistently, we could not detect any effect of treatment on the maximum number of eggs recorded in the colonies (ANOVA: χ^2^ = 1.03, p = 0.31; Additional file 1: Figure S1A).

**Table 1:** Parameters of the models for egg production over time in the control (n = 23) and treatment (n = 18) colonies

Parameter	Estimate control (± se)	Estimate treatment (± se)	t-value	p-value
a	3.23 ± 0.78	2.95 ± 1.17	−0.24	0.81
b	0.40 ± 0.07	0.12 ± 0.11	1.10	0.27
c	−0.03 ± 0	−0.006 ± 0	−1.52	0.13
d	0.0006 ± 0	0.0001 ± 0	1.63	0.10
e	−0.000004 ± 0	−0.000001 ± 0	−1.56	0.12

The models are based on a quintic function $y = a*x + b*x^{2} + c*x^{3} + d*x^{4} + e*x^{5}$. y stands for the number of eggs, x is the number of days after setup, and a, b, c, d and e are the parameters estimated by the models

**Fig. 2:**
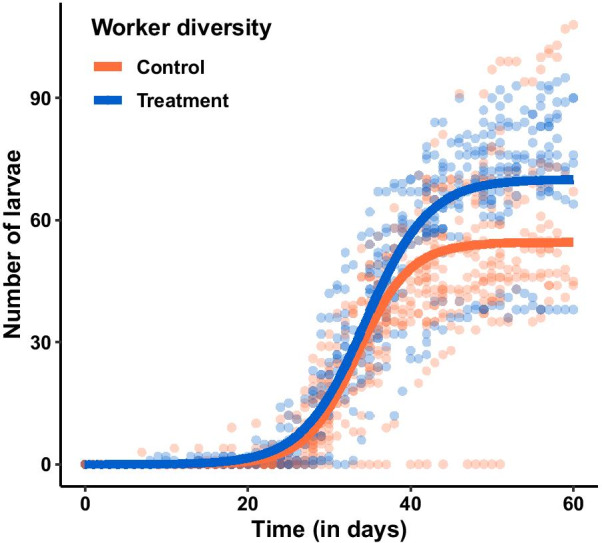
Number of larvae in control (n = 23) and treatment (n = 18) colonies over time. The dots show the raw data for all colonies and time points. The curves depict the output of the models for control (orange) and treatment (blue) colonies. Two parameters of the logistic growth function differed significantly between control and treatment colonies (*Asym*, t = 2.56, p = 0.011; *scal*, t = 2, p = 0.046; Table 2)

The change over time in the number of larvae recorded in the experimental colonies differed significantly between control and treatment colonies (Table 2; Fig. 2). Specifically, we found that colonies with higher worker diversity reached a higher horizontal asymptote by the end of the experiment (*Asym*, t = 2.56, p = 0.011; Table 2; Fig. 2), and showed a higher logistic growth rate (*scal*, t = 2, p = 0.046; Table 2; Fig. 2). We did not detect any effect of worker diversity on the timing of the logistic growth (*xmid*, t = 0.94, p = 0.35; Table 2; Fig. 2).

**Table 2:** Parameters of the models for larva production over time in the control (n = 23) and treatment (n = 18) colonies

Parameter	Estimate control (± se)	Estimate treatment (± se)	t-value	p-value
Asym	54.6 ± 4.0	70.0 ± 6.0	2.56	0.011
xmid	33.5 ± 0.7	34.5 ± 1.0	0.94	0.349
scal	3.24 ± 0.2	3.9 ± 0.3	2.00	0.046

The models are based on a logistic growth function $y = \frac{Asym}{{1 + e^{{\frac{xmid - x}{{scal}}}} }}$. y stands for the number of larvae, x is the number of days after setup, and the parameters estimated by the models are the asymptote (Asym), the timing of the growth (xmid) and the rate of the growth (scal)

In one control colony, no larvae were produced throughout the experiment (Fig. 2). To ensure that the effect of worker diversity on larva production did not stem from this colony only, we repeated the analysis after excluding this colony and found qualitatively similar results, with worker diversity still influencing the level of the asymptote reached at the end of the experiment (*Asym*, t = 2.36, p = 0.018, *xmid*, t = 0.77, p = 0.44, *scal*, t = 1.87, p = 0.061). We also found that the maximum number of larvae recorded in each experimental colony was higher in treatment than in control colonies (ANOVA: χ^2^ = 4.87, p = 0.027, Additional file 1: Figure S1B).

### Experimental increase in worker diversity did not affect foraging

**Fig. 3:**
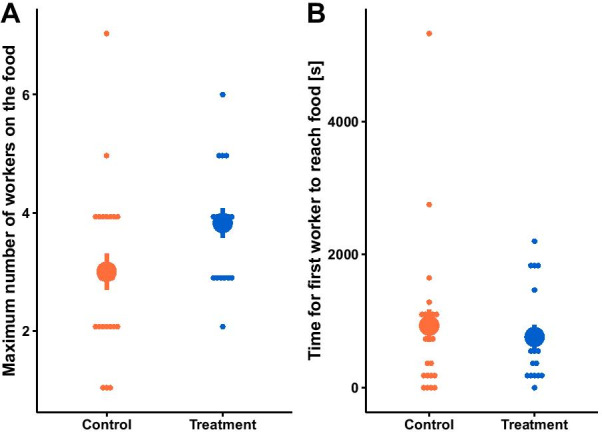
Foraging did not differ between control (n = 23) and treatment colonies (n = 17). The large dots depict the mean ± standard error. The small dots represent the raw data points. We did not detect any significant difference between control and treatment colonies in (**A**) the maximum number of workers (ANOVA: χ^2^ = 2.05, p = 0.15) and (**B**) the time for the first worker to reach the food (ANOVA: χ^2^ = 0.11, p = 0.74)

To investigate whether the beneficial effect of worker diversity on the production of larvae stemmed from improved foraging efficiency, we submitted the experimental colonies to a foraging test. We could not show that control and treatment colonies differed in the maximum number of workers at the food (ANOVA: χ^2^ = 2.05, p = 0.15, Fig. 3A), the proportion of maximum workers at the food (ANOVA: χ^2^ = 1.03, p = 0.31), or the time for the first worker to reach the food (ANOVA: χ^2^ = 0.11, p = 0.74, Fig. 3B).

### Experimental increase in worker diversity enhanced variation in body size

To better understand the positive influence of worker diversity on larva production, we conducted further analyses on the workers that emerged from the pupae that were used to set up the experimental colonies.

**Fig. 4:**
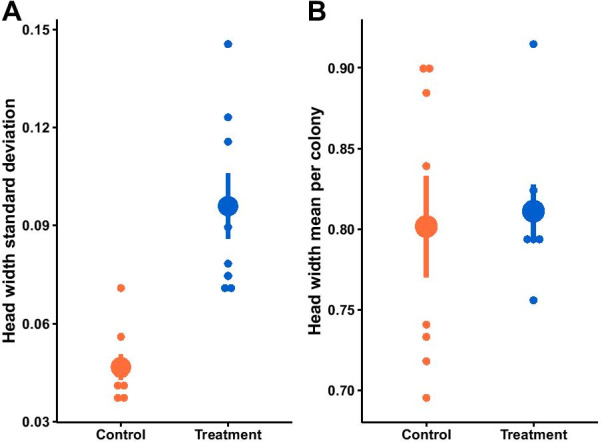
Control (n = 8) and treatment (n = 8) colonies differed in the standard deviation, but not the average, in head width (measured in mm). The large dots depict the mean ± standard error. The small dots represent the raw data points. The experimental increase in worker diversity resulted in (**A**) a higher within-colony variation in head width (ANOVA: F_1,14_ = 26.42, p < 0.001) but (**B**) no difference in the average head width per colony (ANOVA: F_1,14_ = 0.099, p = 0.76)

We extracted the standard deviation in head width for each experimental colony to find that this measure of body size variation differed between control and treatment colonies, as experimental colonies with higher worker diversity showed higher variation (ANOVA: F_1,14_ = 26.42, p < 0.001, Fig. 4A). However, we did not find such an effect of worker diversity on the average mean head width per colony (ANOVA: F_1,14_ = 0.099, p = 0.76, Fig. 4B).

The number of workers that emerged from the pupae used to set up the experimental colonies varied among colonies (20.1 ± 5.4, mean ± sd), but did not differ between control and treatment colonies (ANOVA: χ^2^ = 1.83, p = 0.18). Furthermore, we found that the standard deviation in the time needed for the workers to emerge from the pupae was larger in colonies with higher worker diversity (ANOVA: χ^2^ = 8.7, p = 0.003). We could not detect such an effect of worker diversity on the average time until worker emergence (ANOVA: χ^2^ = 0.003, p = 0.96).

## Discussion

The aim of this study was to test the effect of worker diversity on colony performance in eusocial insects. To do so, we experimentally increased worker diversity of small *Lasius niger* colonies by combining workers from three different source colonies and compared them to colonies composed of workers from a single source colony. In addition, we provided the experimental colonies with unrelated queens to disentangle any effects of worker diversity from maternal effects. We found that increased worker diversity enhanced the production of larvae but not of eggs.

Our finding that worker diversity enhanced larva production in small, experimentally produced laboratory colonies is consistent with previous reports of benefits provided by higher genetic diversity in other species of eusocial Hymenoptera [19, 24, 38, 51, 58–61], a phenomenon termed ‘social heterosis’ [66]. In the honey bee *Apis mellifera*, decreased genetic diversity by artificial insemination, or restricted natural mating was shown to result in lower productivity and fitness [24, 51, 61]. Similarly, in bumble bees (*Bombus terrrestris*) higher genetic variance was shown to decrease parasite load and enhance reproductive success [20, 59, 60]. Our study adds to previous reports because we found benefits of experimentally increasing worker diversity in a species with lower natural levels of diversity, while controlling for any maternal effects caused by the experimental manipulation. Our study validates the hypothesis that worker diversity positively affects larva production, and possibly colony performance.

Worker diversity may improve colony performance via a more efficient division of labor [44]. Behavioral division of labor among workers likely stems from workers differing in their response thresholds to perform specific tasks [34]. In our study, the experimental colonies produced with workers from three source colonies were more diverse than the control colonies in terms of genetic background and size variation. Genetic effects on worker size and morphology have been reported in multiple species of ants [67–69] and worker size differs among colonies in *L. niger* [70]. Worker size and genetic background influence the response threshold of individual workers [30, 67, 71–73], and worker size polymorphism is generally associated with improved division of labor [74–76] (but there were contradictory results [77, 78]). In our study, worker emergence in high diversity colonies was more spread over time, possibly improving division of labor via a broader age distribution [79]. Overall, a more diverse worker force would have resulted in a more heterogeneous mix of response thresholds, possibly enhancing the efficiency of division of labor among workers [38–41, 80–82].

The beneficial effect of worker diversity on the production of larvae likely stemmed from more efficient brood care. We detected an increase in the number of larvae, but not in the number of eggs, and we could not detect any effect on foraging. This suggests that increased worker diversity improved the survival and/or development of larvae, but probably not via better food provisioning to the colony. It may be that the actual distribution of food to the brood was improved by increased worker diversity or that our experiment failed to detect a difference in foraging because of the low number of foragers. However, if more diverse colonies were better at foraging for food, we would have expected the better nourished queens in those colonies to produce more eggs. Our results do not support this expectation. Another explanation that could explain our inability to detect a difference in egg number is egg cannibalism by the larvae, which is common in eusocial Hymenoptera [2, 83, 84]. More diverse colonies had more larvae, which in turn may have eaten more eggs compared to low diversity colonies. According to this scenario, the number of eggs would differ between more and less diverse colonies before the first larva emerged from the eggs. This was not the case, as the maximum number of eggs—which was reached before larvae appeared—was not affected by worker diversity. Our findings are more consistent with worker diversity improving brood care, although we do not have direct evidence for such an effect.

Another, non-mutually exclusive explanation for the enhanced brood production is that more diverse experimental colonies were better at resisting diseases and parasites. This is supported by studies in wasps, bees and ants that reported a positive association between genetic diversity and pathogen resistance [19–27].

A minor proportion of previous studies did not detect an association between diversity and colony performance [51, 61, 62, 64, 65, 81, 82, 85] or had ambiguous findings [58], including in *L. niger* field colonies [49, 65]. Discrepancies among studies suggest that the effect of diversity on division of labor and colony fitness is context- and/or species-dependent. In our study, we used an experimental approach to control for confounding factors and study the benefits of worker diversity in small experimental colonies with young queens, thus in conditions that resemble the early stage of a colony life. Newly founded colonies are very vulnerable and subject to strong competition, and only a small proportion of founding queens manage to establish colonies [2, 86, 87]. Our findings indicate that in this context, increasing worker diversity enhances brood production, which may provide a competitive advantage and increase the chances of a successful colony foundation [87].

The experimental colonies in the high diversity treatment were composed of workers produced by different queens. This situation resembles founding colonies established by several cooperating, unrelated founding queens. This process, called pleometrosis, has been described in multiple ant species, including *L. niger* [88–93]. Pleometrosis increases and accelerates brood production [88, 90, 91, 93–96], which is consistent with our finding that higher worker diversity enhances the production of larvae. Furthermore, workers of young *L. niger* colonies may raid and steal the brood from other colonies in the founding stage [88–91, 93]. Such social parasitism could increase worker diversity in a similar manner to the experimental manipulations conducted in our study, and could similarly benefit colony growth and performance.

We did not manipulate genetic diversity directly, but combined workers from multiple source colonies to produce diversity in the worker force. We confirmed that our experimental manipulation increased size variation in the more diverse colonies. Such size diversity could stem from genetic differences across colonies, but could also be explained by environmental and maternal effects [67, 68, 70, 97]. The genetic background affects size and morphology in eusocial insects [67–69], thus one strategy to increase worker size diversity is to increase genetic diversity. Additionally, we found that the high diversity colonies showed a higher variance in the time of worker emergence from the pupae used to set up the experimental colonies. This result is consistent with the higher variation in worker size, as size and developmental time are correlated [98, 99]. The variation in size and developmental time could also be explained by genetic or source colony effects, as well as other indirect effects of the social context experienced as larvae [100].

So far, evidence for benefits of worker diversity in eusocial insects came from correlative studies, experimental studies where low diversity was also the unnatural situation and/or where other confounding factors such as maternal effects could have played a role. In this study, we experimentally increased worker diversity and controlled for maternal effects. We found that increased worker diversity improved larva production, possibly via enhanced division of labor. Our findings confirm that increased diversity can benefit colony performance in some situations, which could have led to the evolution in some eusocial insects of multiply mated queens and multiple-queen colonies [5, 11, 56, 101].

## Methods

### *Lasius niger* as a study system

To manipulate genetic diversity in the worker force, we used the black garden ant *L. niger* to experimentally combine workers from one or three source colonies and provided them with an unrelated queen. *L. niger* colonies have a single queen, which in Northwestern Europe is usually mated with a single male, leading to highly relatedness among workers [102]. Queens in this species can also be mated twice or more, but mostly in other geographic regions [18, 65]. Established colonies in the field are large, with as much as 10,000 workers [103], which makes it easy to collect large quantities of brood. After their nuptial flights in summer, hundreds of young mated queens can easily be collected as they roam on the ground looking for a nest site [103].

### Collection and housing

We collected 44 *L. niger* queens after their nuptial flight on July 10th 2019 on the campus of Johannes Gutenberg University of Mainz, Germany. One day after collection, we transferred each queen to a glass tube half filled with water blocked by cotton and closed with another piece of cotton. Then, we kept the queens in darkness at 21 °C and approximately 80% humidity and without food, as *L. niger* founding queens do not feed [104]. These queens had produced a first cohort of at least five workers by the time the experiments began.

We collected workers and brood from nine different *L. niger* colonies in the area around the Opel Arena stadium in Mainz, Germany between October and December 2019. The species was identified according to Seifert [105]. In the laboratory, we relocated workers and brood from the soil into glass tubes with water blocked by cotton and covered with aluminum foil. Workers and brood from the same colony were stored in closed boxes (31 × 22 × 5 cm) coated with fluon in a climate cabinet at 28 °C and approximately 100% humidity, and fed five times a week with frozen crickets and a mixture of honey, eggs and vitamins [106]. At the time of collection, these colonies (hereafter referred to as “source colonies”) contained 682 ± 414 (mean ± sd) larvae. We regularly checked the source colonies for pupae to be used for the setup of experimental colonies. All source colonies contributed to both types of experimental colonies (Additional file 1: Table S1).

### Setup of experimental colonies

To manipulate worker diversity, we grouped workers produced by either one or multiple source colonies. Because *L. niger* workers are aggressive towards workers from other colonies, we combined pupae, rather than adult workers, from one or multiple source colonies. The workers that later emerged from those pupae produced the experimental colonies used in this study.

The low diversity experimental colonies were produced by combining 30 pupae from a single source colony and are thus referred to as “control” colonies. We produced the high diversity experimental colonies (hereafter referred to as “treatment” colonies) by combining 30 pupae from three different source colonies (10 pupae per colony). For each experimental colony, we combined the 30 pupae with one unrelated, founding queen and five of its workers (hereafter referred to as “chaperones”) to care for the pupae. For each experimental colony, we removed those chaperones, as well as all the eggs present at the time, once three workers had emerged from the pupae. This day was considered as day 0 in the analysis. Most workers that composed the experimental colonies survived until the end of the monitoring (94.8% ± 8%, mean ± sd), and survival did not differ between control and treatment colonies (Wilcoxon test: W = 214, p = 0.85).

We kept the experimental colonies in closed plastic boxes (11 × 15 × 3 cm) coated with fluon, which contained a glass tube filled with water and cotton as a nest and water source, and a small petri dish for food. We fed the experimental colonies twice a week with a mixture of honey, eggs and vitamins [106]. From day 0 to day 2, we kept the experimental colonies in a dark climate cabinet at approximately 28 °C and 100% humidity. On day 3, we moved the experimental colonies to a climate chamber at 21 °C and approximately 80% humidity and in dark conditions.

In total, we set up 43 colonies. We excluded two treatment colonies from our monitoring because no workers emerged or survived the experimental setup. This resulted in 23 control and 18 treatment colonies in the analysis.

### Brood production monitoring

In each experimental colony, we monitored brood production by counting the number of eggs, larvae and pupae five times a week for 70 days after colony setup. Because the experimental colonies varied in the time to reach day 0 (9 ± 6.6, median ± sd), we only kept the time points between day 0 and day 60 in the analysis to ensure that at any given time point, more than half the experimental colonies were monitored. By the end of the monitoring, only 14 out of 41 colonies had pupae, and even those had a low number of pupae (1.9 ± 2, mean ± sd), and no workers had emerged from the eggs produced in the experimental colonies. Thus, we restricted our analysis of brood production to the production of eggs and larvae.

### Foraging assays

We performed the foraging assays 28 days after the last pupa was observed in the experimental colonies to limit age differences across colonies. One treatment colony was not tested because it still contained two pupae at the end of the experiment. Five days prior to the foraging assays, we removed the food to increase the motivation of workers to forage. We performed the foraging assays inside the box of the experimental colonies by placing a small petri dish with a small cotton roll soaked with a honey solution (0.5 ml honey in 1 ml water). Then we observed the colonies for two hours to score the maximum number of workers observed at the food source at any given time point and to record the time when the first worker arrived at the food.

### Body size measurements

At the end of the experiments, all workers from the experimental colonies were frozen at −18 °C for later morphological measurements. To estimate body size, we measured the width of the worker heads as the distance between the outer points of the eyes [78, 85, 107, 108]. The frozen workers were placed flatly on modeling clay, photographed with a Leica S9i microscope, and measured with LAS V4.12 Leica computer software. We measured all 326 workers that survived the experiment in eight control (151 workers) and eight treatment (175 workers) colonies, while making sure that we only used one experimental colony per source colony.

### Statistical analysis

To test whether control and treatment colonies differed in brood production over time, we built non-linear mixed effect models with the R package *nlme* [109].

To model the egg production, we used the quintic function

$$y = a{*}x + b{*}x^{2} + c{*}x^{3} + d{*}x^{4} + e{*}x^{5}$$

where *y* is the number of eggs, *x* is the number of days, and the parameters *a*, *b*, *c*, *d* and *e* are estimated by the model to provide the best fit to the empirical data.

To model the larva production, we used a logistic growth equation with the SSlogis() function

$$y = \frac{Asym}{{1 + e^{{\frac{xmid - x}{{scal}}}} }}$$

where *y* is the number of larvae, *x* is the number of days, and the parameters *Asym*, *xmid* and *scal* are estimated by the model to provide the best fit to the empirical data. *Asym* represents the horizontal asymptote of the logistic growth function, *xmid* the x value of the sigmoid’s midpoint, and *scal* the rate of the logistic growth. The starting values for the parameters were obtained by fitting non-linear models without random effects using the nls() function. For both eggs and larvae, the non-linear mixed effects models were fitted using the function nlme(), and included the worker diversity (control or treatment) as fixed effect and the experimental colony as random effect. The summary() function was used to extract the estimate for each parameter and each treatment, as well as to test whether estimates differed between treatments. In addition to the non-linear modeling of the change in brood number over time, we used the simpler approach of extracting the maximum number of eggs and larvae recorded in each colony during the experiment. This allowed us to confirm that any effect that would be detected by the non-linear models would not merely stem from the source colony not being included as random effect in the models. We then tested the effect of worker diversity on the maximum numbers of brood using the lmer() function with the package *lme4* [110] to fit a linear mixed effects model with worker diversity as fixed effect and source colony as random effect.

We tested the effect of worker diversity on the maximum number of foragers at the food source, as well as the square root transformed data of the time for the first worker to reach the food by building a linear mixed effect model using the lmer() function*,* with worker diversity as fixed effect and source colony as random effect.

To test whether there was a difference in the size of workers used to set up the control and treatment colonies, we calculated the square root of the average head width per colony. To investigate the effect of worker diversity on size variation, we extracted the standard deviation of head size in each colony. Then we built linear models with the lm() function to explain both measurements by worker diversity.

We tested the effect of worker diversity on the number of workers that emerged from the pupae used to set up the experimental colonies, and the standard deviation and mean of the time needed for the workers to emerge with linear mixed effect models using the lmer() function*,* with worker diversity as fixed effect and source colony as random effect. We checked all linear models for normal distribution of the residuals and used the Anova() function of the package *car* [111] to test the effect of the explanatory variables. We produced all plots with the packages *ggplot2* [112] and *ggpubr* [113], and used the package *dplyr* [114] for data handling. We ran all analyses in R [115] version 3.6.1. The R script is provided in Additional file 2, and all data in Additional file 3.

## Supplementary Information

**Additional file 1. Figure S1**. Maximum number of brood items in the different treatments.** Table S1**. Number of control and treatment colonies that have been created by the respective source colonies.

**Additional file 2.** The R script for all statistical analyses.

**Additional file 3.** All data generated and analyzed in this study.

## Data Availability

All data generated or analyzed during this study are included in Additional file 3.

## References

[1] Hamilton WD (1964). The genetical evolution of social behaviour. I J Theor Biol.

[2] Hölldobler B, Wilson EO (1990). The ants.

[3] Hughes WOH, Oldroyd BP, Beekman M, Ratnieks FLW (2008). Ancestral monogamy shows kin selection is key to the evolution of Eusociality. Science..

[4] Boomsma JJ (2009). Lifetime monogamy and the evolution of eusociality. Philos Trans R Soc B Biol Sci.

[5] Hughes WOH, Ratnieks FLW, Oldroyd BP (2008). Multiple paternity or multiple queens: two routes to greater intracolonial genetic diversity in the eusocial Hymenoptera. J Evol Biol.

[6] Tarpy DR, Caren JR, Delaney DA, Sammataro D, Finley J, Loper GM (2010). Mating frequencies of Africanized honey bees in the south western USA. J Apic Res.

[7] Tarpy DR, Nielsen R, Nielsen DI (2004). A scientific note on the revised estimates of effective paternity frequency in Apis. Insectes Soc.

[8] Tarpy DR, Delaney DA, Seeley TD (2015). Mating frequencies of honey bee queens (*Apis mellifera* l.) in a population of feral colonies in the Northeastern United States. PLoS One..

[9] Adams J, Rothman ED, Kerr WE, Paulino ZL (1977). Estimation of the number of sex alleles and queen matings from diploid male frequencies in a population of *Apis mellifera*. Genetics.

[10] Ingram KK (2002). Flexibility in nest density and social structure in invasive populations of the Argentine ant, *Linepithema humile*. Oecologia.

[11] Palmer KA, Oldroyd BP (2000). Evolution of multiple mating in the genus Apis. Apidologie.

[12] Baer B, Armitage SAO, Boomsma JJ (2006). Sperm storage induces an immunity cost in ants. Nature.

[13] Hamilton WD (1967). Extraordinary sex ratios. Science..

[14] Boomsma JJ, Grafen A (1990). Intraspecific variation in ant sex ratios and the Trivers-Hare Hypothesis. Evolution (N Y).

[15] Strassmann JE (1984). Female-biased sex ratios in social insects lacking morphological castes. Evolution (N Y).

[16] Mehdiabadi NJ, Reeve HK, Mueller UG (2003). Queens versus workers: sex-ratio conflict in eusocial Hymenoptera. Trends Ecol Evol.

[17] Crozier RH, Fjerdingstad EJ (2001). Polyandry in social hymenoptera - disunity in diversity ?. Finnish Zool Bot Publ Board.

[18] Corley M, Fjerdingstad EJ (2011). Mating strategies of queens in *Lasius niger* ants—is environment type important?. Behav Ecol Sociobiol.

[19] Shykoff JA, Schmid-Hempel P (1991). Parasites and the advantage of genetic variability within social insect colonies. Proc R Soc B Biol Sci.

[20] Liersch S, Schmid-Hempel P (1998). Genetic variation within social insect colonies reduces parasite load. Proc R Soc B Biol Sci.

[21] Ugelvig LV, Kronauer DJC, Schrempf A, Heinze J, Cremer S (2010). Rapid anti-pathogen response in ant societies relies on high genetic diversity. Proc R Soc B Biol Sci.

[22] Tarpy DR, Seeley TD (2006). Lower disease infections in honeybee (*Apis mellifera*) colonies headed by polyandrous vs monandrous queens. Naturwissenschaften.

[23] Reber A, Castella G, Christe P, Chapuisat M (2008). Experimentally increased group diversity improves disease resistance in an ant species. Ecol Lett.

[24] Simone-Finstrom M, Walz M, Tarpy DR (2016). Genetic diversity confers colony-level benefits due to individual immunity. Biol Lett.

[25] Tarpy DR (2003). Genetic diversity within honeybee colonies prevents severe infections and promotes colony growth. Proc R Soc B Biol Sci.

[26] Saga T, Okuno M, Loope KJ, Tsuchida K, Ohbayashi K, Shimada M (2020). Polyandry and paternity affect disease resistance in eusocial wasps. Behav Ecol.

[27] Hughes WOH, Boomsma JJ (2004). Genetic diversity and disease resistance in leaf-cutting ant societies. Evolution (N Y).

[28] Modlmeier AP, Foitzik S (2011). Productivity increases with variation in aggression among group members in Temnothorax ants. Behav Ecol.

[29] Constant N, Santorelli LA, Lopes JFS, Hughes WOH (2012). The effects of genotype, caste, and age on foraging performance in leaf-cutting ants. Behav Ecol.

[30] Waddington SJ, Santorelli LA, Ryan FR, Hughes WOH (2010). Genetic polyethism in leaf-cutting ants. Behav Ecol.

[31] Eyer PA, Freyer J, Aron S (2013). Genetic polyethism in the polyandrous desert ant *Cataglyphis cursor*. Behav Ecol.

[32] Julian GE, Fewell JH (2004). Genetic variation and task specialization in the desert leaf-cutter ant, *Acromyrmex versicolor*. Anim Behav.

[33] Libbrecht R, Keller L (2013). Genetic compatibility affects division of labor in the Argentine ant *Linepithema humile*. Evolution (N Y).

[34] Robinson GE (1987). Regulation of honey bee age polyethism by juvenile hormone. Behav Ecol Sociobiol.

[35] Beshers SN, Fewell JH (2001). Models of division of labor in social insects. Annu Rev Entomol.

[36] Oldroyd BP, Fewell JH (2007). Genetic diversity promotes homeostasis in insect colonies. Trends Ecol Evol.

[37] Johnson BR (2010). Spatial effects, sampling errors, and task specialization in the honey bee. Insectes Soc.

[38] Jones JC, Myerscough MR, Graham S, Oldroyd BP (2004). Honey bee nest thermoregulation: diversity promotes stability. Science..

[39] Stuart RJ, Page RE (1991). Genetic component to division of labor among workers of a leptothoracine ant. Naturwissenschaften.

[40] Blatrix R, Durand JL, Jaisson P (2000). Task allocation depends on matriline in the ponerine ant *Gnamptogenys striatula* Mayr. J Insect Behav.

[41] Breed MD, Rogers KB (1991). The behavioral genetics of colony defense in honeybees: genetic variability for guarding behavior. Behav Genet.

[42] Ulrich Y, Kawakatsu M, Tokita CK, Saragosti J, Chandra V, Tarnita CE (2021). Response thresholds alone cannot explain empirical patterns of division of labor in social insects. PLoS Biol.

[43] Jeanson R, Weidenmüller A (2014). Interindividual variability in social insects - proximate causes and ultimate consequences. Biol Rev.

[44] Smith CR, Toth AL, Suarez AV, Robinson GE (2008). Genetic and genomic analyses of the division of labour in insect societies. Nat Rev Genet.

[45] Schluns E, Wegener B, Robson S (2011). Genetic polyethism and nest building in the weaver ant *Oecophylla smaragdina* (Fabricius, 1775)(Hymenoptera: Formicidae). Myrmecological News.

[46] Crozier RH, Page RE (1985). On being the right size: male contributions and multiple mating in social Hymenoptera. Behav Ecol Sociobiol.

[47] Boomsma JJ, Ratnieks FLW (1996). Paternity in eusocial Hymenoptera. Philos Trans R Soc London Ser B Biol Sci.

[48] Gove R, Hayworth M, Chhetri M, Rueppell O (2009). Division of labour and social insect colony performance in relation to task and mating number under two alternative response threshold models. Insectes Soc.

[49] Fjerdingstad EJ, Keller L (2004). Relationships between phenotype, mating behavior, and fitness of queens in the ant *Lasius niger*. Evolution (N Y).

[50] Fjerdingstad EJ, Gertsch PJ, Keller L (2002). Why do some social insect queens mate with several males? Testing the sex-ratio manipulation hypothesis in *Lasius niger*. Evolution (N Y).

[51] Mattila HR, Seeley TD (2007). Genetic diversity in honey bee colonies enhances productivity and fitness. Science..

[52] Wiernasz DC, Perroni CL, Cole BJ (2004). Polyandry and fitness in the western harvester ant, *Pogonomyrmex occidentalis*. Mol Ecol.

[53] Pedersen JS, Boomsma JJ (1999). Positive association of queen number and queen-mating frequency in Myrmica ants: a challenge to the genetic-variability hypotheses. Behav Ecol Sociobiol.

[54] Cole BJ, Wiernasz DC (1999). The selective advantage of low relatedness. Science..

[55] Dobelmann J, Loope KJ, Wilson-Rankin E, Quinn O, Baty JW, Gruber MAM (2017). Fitness in invasive social wasps: the role of variation in viral load, immune response and paternity in predicting nest size and reproductive output. Oikos.

[56] Goodisman MAD, Kovacs JL, Hoffman EA (2007). The significance of multiple mating in the social wasp *Vespula maculifrons*. Evolution (N Y).

[57] Loope KJ, Chien C, Juhl M (2014). Colony size is linked to paternity frequency and paternity skew in yellowjacket wasps and hornets. BMC Evol Biol.

[58] Oldroyd BP, Rinderer TE, Harbo JR, Buco SM (1992). Effects of intracolonial genetic diversity on honey bee (Hymenoptera: Apidae) colony performance. Ann Entomol Soc Am.

[59] Baer B, Schmid-Hempel P (2001). Unexpected consequences of polyandry for parasitism and fitness in the bumblebee, *Bombus terrestris*. Evolution (N Y).

[60] Baer B, Schmid-Hempel P (1999). Experimental variation in polyandry affects parasite loads and fitness in a bumble-bee. Nature.

[61] Fuchs S, Schade V (1994). Lower performance in honeybee colonies of uniform paternity. Apidologie.

[62] Ulrich Y, Saragosti J, Tokita CK, Tarnita CE, Kronauer DJC (2018). Fitness benefits and emergent division of labour at the onset of group living. Nature.

[63] Trontti K, Thurin N, Sundström L, Aron S (2007). Mating for convenience or genetic diversity? Mating patterns in the polygynous ant *Plagiolepis pygmaea*. Behav Ecol.

[64] Pearcy M, Timmermans I, Allard D, Aron S (2009). Multiple mating in the ant *Cataglyphis cursor*: testing the sperm limitation and the diploid male load hypotheses. Insectes Soc.

[65] Fjerdingstad EJ, Gertsch PJ, Keller L (2003). The relationship between multiple mating by queens, within-colony genetic variability and fitness in the ant *Lasius niger*. J Evol Biol.

[66] Nonacs P, Kapheim KM (2007). Social heterosis and the maintenance of genetic diversity. J Evol Biol.

[67] Hughes WOH, Sumner S, Van Borm S, Boomsma JJ (2003). Worker caste polymorphism has a genetic basis in Acromyrmex leaf-cutting ants. Proc Natl Acad Sci U S A.

[68] Schwander T, Rosset H, Chapuisat M (2005). Division of labour and worker size polymorphism in ant colonies: the impact of social and genetic factors. Behav Ecol Sociobiol.

[69] Friedman DA, Gordon DM (2016). Ant genetics: reproductive physiology, worker morphology, and behavior. Annu Rev Neurosci.

[70] Grześ IM, Okrutniak M, Gorzałczany M, Piszczek P (2019). Body size variation of the ant *Lasius niger* along a metal pollution gradient. Environ Sci Pollut Res.

[71] Uribe-Rubio JL, Guzmán-Novoa E, Vázquez-Peláez CG, Hunt GJ (2008). Genotype, task specialization, and nest environment influence the stinging response thresholds of individual Africanized and European honeybees to electrical stimulation. Behav Genet.

[72] Rheindt FE, Strehl CP, Gadau J (2005). A genetic component in the determination of worker polymorphism in the Florida harvester ant *Pogonomyrmex badius*. Insectes Soc.

[73] Hughes WOH, Boomsma JJ (2007). Genetic polymorphism in leaf-cutting ants is phenotypically plastic. Proc R Soc B Biol Sci.

[74] Cerdá X, Retana J, Cerda X (1997). Links between worker polymorphism and thermal biology in a thermophilic ant species. Oikos.

[75] Beshers SN, Traniello JFA (1996). Polyethism and the adaptiveness of worker size variation in the attine ant *Trachymyrmex septentrionalis*. J Insect Behav.

[76] Huang MH (2010). Multi-phase defense by the big-headed ant, *Pheidole obtusospinosa*, against raiding army ants. J Insect Sci.

[77] Honorio R, Doums C, Molet M (2020). Manipulation of worker size diversity does not affect colony fitness under natural conditions in the ant *Temnothorax nylanderi*. Behav Ecol Sociobiol..

[78] Colin T, Doums C, Péronnet R, Molet M (2017). Decreasing worker size diversity does not affect colony performance during laboratory challenges in the ant *Temnothorax nylanderi*. Behav Ecol Sociobiol.

[79] Enzmann BL, Nonacs P (2021). Age-related division of labor occurs in ants at the earliest stages of colony initiation. Behav Ecol Sociobiol..

[80] Ranger S, O’Donnell S (1999). Genotypic effects on forager behavior in the neotropical stingless bee *Partamona bilineata* (Hymenoptera: *Meliponidae*). Naturwissenschaften.

[81] O’Donnel S (1998). Genetic effects on task performance, but not on age polyethism, in a swarm-founding eusocial wasp. Anim Behav.

[82] Page RE, Robinson GE, Fondrk MK, Nasr ME (1995). Effects of worker genotypic diversity on honey bee colony development and behavior (*Apis mellifera* L.). Behav Ecol Sociobiol..

[83] Schultner E, D’Ettorre P, Helanterä H (2013). Social conflict in ant larvae: egg cannibalism occurs mainly in males and larvae prefer alien eggs. Behav Ecol.

[84] Urbani CB (1991). Indiscriminate oophagy by ant larvae: an explanation for brood serial organization?. Insectes Soc.

[85] Fournier D, Battaille G, Timmermans I, Aron S (2008). Genetic diversity, worker size polymorphism and division of labour in the polyandrous ant *Cataglyphis cursor*. Anim Behav.

[86] Whitcomb WH, Bhatkar A, Nickerson JC (1973). Predators of Solenopsis invicta queens prior to successful colony establishment. Environ Entomol.

[87] Tschinkel WR (1992). Brood raiding in the fire ant, *Solenopsis invicta* (Hymenoptera: Formicidae): laboratory and lield observations. Ann Entomol Soc Am.

[88] Trunzer B, Heinze J, Hölldobler B (1998). Cooperative colony founding and experimental primary polygyny in the ponerine ant *Pachycondyla villosa*. Insectes Soc.

[89] Rissing SW, Pollock GB (1987). Queen aggression, pleometrotic advantage and brood raiding in the ant *Veromessor pergandei* (Hymenoptera: Formicidae). Anim Behav.

[90] Deslippe RJ, Savolainen R (1995). Colony foundation and polygyny in the ant *Formica podzolica*. Behav Ecol Sociobiol.

[91] Johnson RA (2004). Colony founding by pleometrosis in the semiclaustral seed-harvester ant *Pogonomyrmex californic*us (Hymenoptera: Formicidae). Anim Behav.

[92] Wheeler WM (1917). The pleometrosis of Myrmecocystus. Psyche A J Entomol.

[93] Sommer K, Hölldobler B (1995). Colony founding by queen association and determinants of reduction in queen number in the ant *Lasius niger*. Anim Behav.

[94] Teggers EM, Deegener F, Libbrecht R (2021). Fecundity determines the outcome of founding queen associations in ants. Sci Rep.

[95] Madsen NEL, Offenberg J (2017). Effect of pleometrosis and brood transplantation on colony growth of the black garden ant, *Lasius niger*. Asian Myrmecol.

[96] Waloff N (1957). The effect of the number of queens of the ant *Lasius flavus* (Fab.) (Hym., Formicidae) on their survival and on the rate of development of the first brood. Insectes Soc..

[97] Wills BD, Powell S, Rivera MD, Suarez AV (2018). Correlates and consequences of worker polymorphism in ants. Annu Rev Entomol.

[98] Teder T, Vellau H, Tammaru T (2014). Age and size at maturity: a quantitative review of diet-induced reaction norms in insects. Evolution (N Y).

[99] Atkinson D (1994). Temperature and organism size—a biological law for ectotherms?. Adv Ecol Res.

[100] Tripet F, Nonacs P (2004). Foraging for work and age-based polyethism: the roles of age and previous experience on task choice in ants. Ethology.

[101] Cole BJ (1983). Multiple mating and the evolution of social behavior in the Hymenoptera. Behav Ecol Sociobiol.

[102] Boomsma JJ, Van Der Have TM (1998). Queen mating and paternity variation in the ant *Lasius niger*. Mol Ecol.

[103] Parker JD, Parker KM (2006). Ants as naturally long-lived insect models for aging.

[104] Keller L, Passera L (1989). Size and fat content of gynes in relation to the mode of colony founding in ants (Hymenoptera; Formicidae). Oecologia.

[105] Seifert B. The ants of central and north Europe. lutra Verlags-und Vertriebsgesellschaft; 2007.

[106] Bhatkar A, Whitcomb WH (1970). Artificial diet for rearing various species of ants. Florida Entomol.

[107] Okrutniak M, Rom B, Turza F, Grześ IM (2020). Body size differences between foraging and intranidal workers of the monomorphic ant *Lasius niger*. Insects.

[108] Tschinkel WR, Mikheyev AS, Storz SR (2003). Allometry of workers of the fire ant, *Solenopsis invicta*. J Insect Sci..

[109] Pinheiro J, Bates D, DebRoy S, Sarkar D, R Core Team. {nlme}: Linear and nonlinear mixed effects models. 2020. https://cran.r-project.org/package=nlme.

[110] Bates D, Mächler M, Bolker B, Walker S (2015). Fitting linear mixed-effects models using lme4. J Stat Softw..

[111] Fox J, Weisberg S. An {R} companion to applied regression. Third. Thousand Oaks {CA}: Sage; 2019. https://socialsciences.mcmaster.ca/jfox/Books/Companion/.

[112] Wickham H. ggplot2: Elegant graphics for data analysis. 2016. https://ggplot2.tidyverse.org.

[113] Kassambara A. ggpubr: “ggplot2” based publication ready plots. 2020. https://cran.r-project.org/package=ggpubr.

[114] Wickham H, François R, Henry L, Müller K. dplyr: A Grammar of data manipulation. 2020. https://cran.r-project.org/package=dplyr.

[115] R Core Team. R: a language and environment for statistical computing. 2020. https://www.r-project.org/.

