# Supplementary material for: Experimental increase of worker diversity benefits brood production in ants
Source: BMC Ecol Evol. 2021 Aug 30;21:163. doi: 10.1186/s12862-021-01890-x (PMC8404329; doi:10.1186/s12862-021-01890-x)
Supplement: Supplementary file 1 — Additional file 1. Figure S1. Maximum number of brood items in the different treatments. Table S1. Number of control and treatment colonies that have been created by the respective source colonies. [file 12862_2021_1890_MOESM1_ESM.docx]

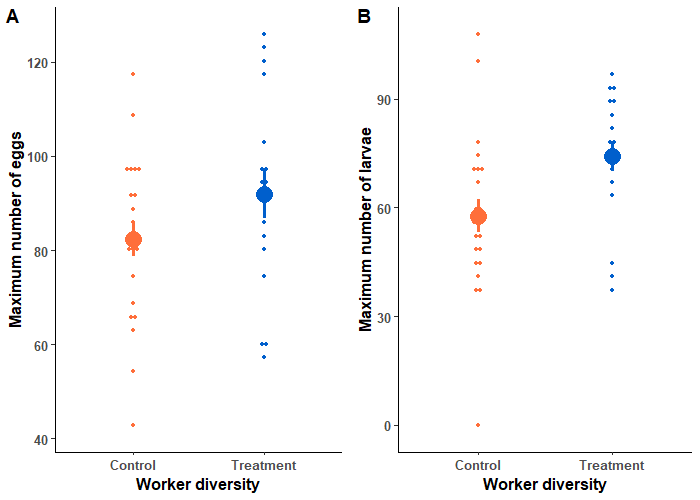


**Figure S1** Maximum number of brood items in the different treatments. Maximum number of (A) eggs and (B) larvae produced by control and treatment colonies within 60 days of monitoring. The large dots depict the mean ± standard error. The small dots represent the raw data points. (A) There is no significant difference between the maximum number of eggs produced between treatments (ANOVA: χ^2^ = 1.03, p = 0.31). (B) The maximum number of larvae between treatment and control colonies was significantly different (ANOVA: χ^2^ = 4.87, p = 0.027).

**Table S1** Number of control and treatment colonies that have been created by the respective source colonies. We created 23 control and 18 treatment colonies from 9 source colonies. The numbers for the treatment colonies do not add to 18 because each treatment colony is made up of three source colonies.

| **Source colony** | **Contributed to this number of control colonies** | **Contributed to this number of treatment colonies** |
| --- | --- | --- |
| A | 2 | 4 |
| B | 3 | 5 |
| C | 3 | 8 |
| D | 2 | 6 |
| E | 2 | 7 |
| F | 2 | 8 |
| G | 3 | 9 |
| H | 4 | 5 |
| I | 2 | 2 |
